# Body composition determinants of radiation dose during abdominopelvic CT

**DOI:** 10.1007/s13244-017-0577-y

**Published:** 2017-10-23

**Authors:** Patrick D. McLaughlin, Liam Chawke, Maria Twomey, Kevin P. Murphy, Siobhán B. O’Neill, Sebastian R. McWilliams, Karl James, Richard G. Kavanagh, Charles Sullivan, Faimee E. Chan, Niamh Moore, Owen J. O’Connor, Joseph A. Eustace, Michael M. Maher

**Affiliations:** 1 Department of Emergency and Trauma Radiology, Vancouver General Hospital, Jim Pattison Pavilion North, #3350-950 West 10th Ave, Vancouver, BC V5Z 1M9 Canada; 2 Department of Radiology, University College Cork, Cork, Ireland; 3 Department of Radiology, Cork University Hospital, Wilton, Cork, Ireland; 4 Department of Nephrology, Cork University Hospital, Cork, Ireland; 5 Health Research Board Clinical Research Facility, University College Cork, Cork, Ireland

**Keywords:** Tomography, X-ray computed, Radiation dosage, Intra-abdominal fat, Muscle, Skeletal, Body mass index

## Abstract

**Objectives:**

We designed a prospective study to investigate the in-vivo relationship between abdominal body composition and radiation exposure to determine the strongest body composition predictor of dose length product (DLP) at CT.

**Methods:**

Following institutional review board approval, quantitative analysis was performed prospectively on 239 consecutive patients who underwent abdominopelvic CT. DLP, BMI, volumes of abdominal adipose tissue, muscle, bone and solid organs were recorded.

**Results:**

All measured body composition parameters correlated positively with DLP. Linear regression (R^2^ = 0.77) revealed that total adipose volume was the strongest predictor of radiation exposure [B (95% CI) = 0.027(0.024–0.030), t=23.068, *p* < 0.001]. Stepwise linear regression using DLP as the dependent and BMI and total adipose tissue as independent variables demonstrated that total adipose tissue is more predictive of DLP than BMI [B (95% CI) = 16.045 (11.337-20.752), t=6.681, *p* < 0.001].

**Conclusions:**

The volume of adipose tissue was the strongest predictor of radiation exposure in our cohort.

***Main message*:**

• *Individual body composition variables correlate with DLP at abdominopelvic CT.*

• *Total abdominal adipose tissue is the strongest predictor of radiation exposure.*

• *Muscle volume is also a significant but weaker predictor of DLP.*

## Introduction

At present there is a considerable research and industry drive to reduce the radiation dose during CT scanning while preserving image quality and diagnostic accuracy. To date, CT dose reduction technology including automated tube current modulation and iterative reconstruction have facilitated reductions in CT dose to levels approximately 70–75% less than what they were a decade ago. Larger reductions in dose are conceivable with continued research and development and more recent advances in CT technology have facilitated significant dose reductions without sacrificing image quality [1–3].

Differences in patient size and body weight challenge the pathways of CT dose reduction. It is well recognised that patients with a larger body habitus are exposed to significantly larger doses of ionising radiation during abdominopelvic CT when automated tube current modulation (ATCM) is employed [4–7]. Previous studies have examined the influence of variables such as body weight [6], body mass index [4], patient cross-sectional area [5, 7] and patient AP diameter [8] on imparted dose during abdominopelvic CT with ATCM. However, the abdominal compartment houses many structures of varying volume and density, which impact these indices. These constituents include the solid abdominal organs, soft tissue structures such as abdominal musculature and adipose tissue and bony structures such as the lumbar spine and pelvis. These structures all contribute to patient body weight, body mass index and cross-sectional area and are therefore also likely to contribute individually to the imparted dose during abdominopelvic CT. Although one previous study found an association between subjectively graded quantities of abdominal fat and effective dose [9], we found no study that has investigated the in-vivo relationship between multiple abdominal body composition variables and radiation dose during CT with ATCM. The authors believe that further investigation of such factors, which may significantly differ among individuals of similar weight, cross-sectional area and BMI [10], may guide future methods of dose optimisation in abdominopelvic CT and may allow radiologists to refine examination technique and ATCM protocol particularly in obese patients.

We therefore designed a prospective, cross-sectional study with the following aims:

1. To identify the body composition determinants of an elevated dose length product during abdominopelvic CT.
2. To determine which of the following parameters is the strongest predictor of radiation dose at CT:
  Total abdominopelvic adipose tissue volume
  Abdominopelvic muscle volume
  Abdominopelvic bone volume
  Solid organ volumes

## Materials and methods

Following institutional review board approval, 239 consecutive patients who were referred for clinically indicated abdominopelvic CT were prospectively recruited over a 3-month period. All patients were scanned using a single 64-slice multi-detector row CT scanner (General Electric Lightspeed VCT-XTe, GE Healthcare, GE Medical Systems, Milwaukee, WI, USA). Exclusion criteria included patients who were less than 18 years of age, those undergoing CT outside of normal operational hours at our department and those who did not receive intravenous or oral iodinated contrast. The institutional review board waived the requirement for written informed consent. Patients had their weight and height measured using a digital device (Seca electronic measuring station Model 763, Seca Medical, Hamburg, Germany) and their body mass index (BMI) was subsequently recorded.

### CT technique

Each CT scan covered an identical anatomic area extending from the dome of the diaphragm to the pubic symphysis. Each patient received 1L of positive oral contrast (2% Gastrograffin, Bracco Diagnostics Inc., Princeton, NJ) and a 100-ml bolus of intravenous contrast (Iohexol, Omnipaque 300, GE Healthcare, Mississauga, ON) delivered at a flow rate of 2.5 ml/s. Our routine departmental scanning protocol was used in all cases consisting of a tube voltage of 120 kV, rotation time of 0.8 s, pitch of 0.984:1, z-axis automated tube current modulation with minimum and maximum tube current thresholds set at 120 and 300 mA and a noise index of 35.3 HU. Images were acquired at 0.625 mm and subsequently reconstructed to a slice thickness of 2 mm with 40% Adaptive Statistical Iterative Reconstruction (ASIR, GE Healthcare, Milwaukee, WI, USA). Dose length product (DLP) values were recorded from each CT dose report and calibration of the CT unit was performed once per week in accordance with the manufacturer’s instructions. The protocol was not modified to account for patient BMI apart from employment of automated tube current modulation.

### Quantitative analysis

A single reader (LC) analysed every CT data set using a combination of “semi-automated” threshold-based and “manual” region of interest-based quantitative CT techniques. The following body composition parameters were recorded in each patient: total volume of abdominopelvic adipose tissue (TAT), muscle volume (MV), bone volume (BV) and volumes of the solid organs including the liver, both kidneys and spleen. Visceral adipose area (VAA) and subcutaneous adipose area (SAA) were measured on a single slice so that these could be compared with total adipose volume estimates. A second reader (FC) repeated each measurement in a random sample of 30% of the patient cohort (*n* = 71) allowing for calculation of inter-rater agreement. Parameters requiring threshold-based segmentation, e.g. total volume of the abdomen/pelvis, total volume of fat and bone volume, were estimated using a commercial workstation (Advantage Workstation VolumeShare 2, Version 4.4, GE Medical Systems, Milwaukee, WI) and organ volumes were calculated using manually drawn regions of interest with Osirix, an open source medical imaging package (OsiriX Foundation, Geneva Switzerland).

The total volume of adipose tissue was calculated by automatically removing pixels outside the density range of fat (−190 HU to -30 HU) [11] within the imaged range of the abdomen and pelvis. Skeletal muscle volume throughout the scanned range was calculated in a semi-automated fashion using the threshold range of -29 HU to +150 HU [12]. Bone volume was also semi-automatically estimated by performing the “Autobone extract” function with subsequent manual removal of any high-density oral contrast or non-skeletal calcifications using three-dimensional MIP reconstructions and the scissor tool. Both visceral and subcutaneous adipose tissue areas were calculated on a single axial image situated 6 cm superior to the L4–L5 intervertebral space as recommended by Demerath et al. [13, 14]. Abdominopelvic organs were outlined manually on axial CT images using the curved ROI tool in Osirix, a process that was repeated on adjacent slices until the organ was completely delineated.

### Statistical analysis

Statistical analysis was performed using a commercially available medical statistical package (PASW version 20, SPSS Inc., Chicago, IL, USA) (JE, PMcL). Quantitative indices were compared and correlated using the Student’s t- and Mann Whitney U-test and Pearson and Spearman’s tests, respectively. Linear regression analysis with DLP as the dependent variable was used to define the strongest body composition determinant of increased radiation dose. Agreement between the two quantitative CT readers was compared using the intra-class correlation coefficient. A difference with a *p*-value of <0.05 was considered statistically significant. All data are presented as mean ± standard deviation unless otherwise stated. Result variables were grouped by quartile, where appropriate. As body composition is known to vary by both sex and age, a subgroup analysis was performed. The study sample as divided into four subgroups according to patient sex (male, female) and patient age (younger, age < 50th percentile; older, age > 50th percentile) (50th percentile, 59 years) to evaluate these differences and to compare their effect on the radiation dose.

## Results

**Fig. 1:**
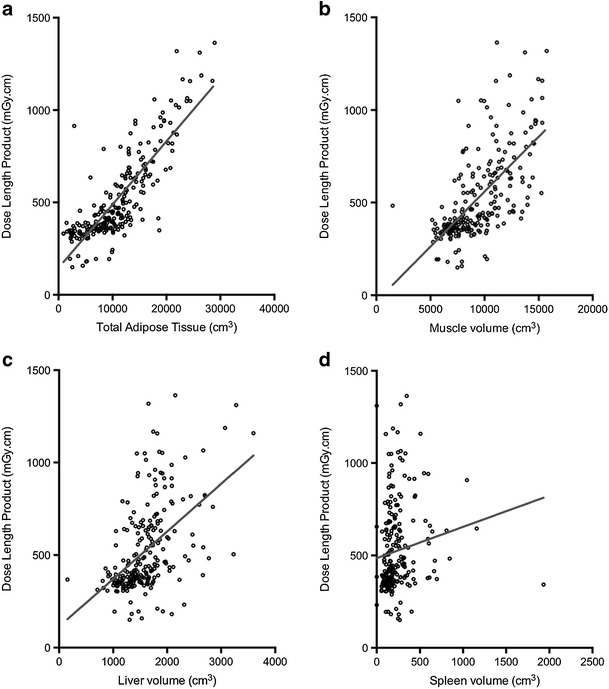
**a** Scatter plot with linear regression line outlining the distribution of total adipose tissue volume (cm^3^) and dose length product (mGy.cm) in our study sample (*n* = 239). **b** Scatter plot with linear regression line outlining the distribution of muscle volume (cm^3^) and dose length product (mGy.cm) in our study sample (n = 239). **c** Scatter plot with linear regression line outlining the distribution of liver volume (cm^3^) and dose length product (mGy.cm) in our study sample (n = 239). **d** Scatter plot with linear regression line outlining the distribution of spleen volume (cm^3^) and dose length product (mGy.cm) in our study sample (n = 239)

A total of 239 patients, 125 females and 114 males with a mean age of 56.6 ± 17.9 years (range, 19–95 years) and a mean BMI of 26.5 ± 5.42 kg/m^2^ (range, 13.9–49.1 kg/m^2^) were included for quantitative analysis. The mean DLP imparted across the study sample was 524 ± 236 mGy.cm (range, 149–1363 mGy.cm). There was a statistically significant correlation between patient BMI and DLP (Pearson’s correlation; *r* = 0.797 *p* < 0.001). DLP was significantly higher in male patients (580 ± 243 vs. 473 ± 219 mGy.cm, p < 0.001) and male patients also had significantly higher BMIs than female patients (28.5 ± 4.8 vs. 24.7 ± 5.3 kg/m^2^). All measured body composition parameters correlated positively with DLP although the correlation was weakest for spleen volume, only reaching statistical significance at the 0.05 level (Table 1) (Fig. 1a–d). Good agreement was found when all body composition variables measured by two observers were compared (intraclass correlation coefficient of group = 0.877, *p* < 0.001, *n* = 71). Agreement was best for total adipose volume estimation between observers (intraclass correlation coefficient of TAT = 0.999, p < 0.001, n = 71).

**Table 1:** Patient BMI and body composition variables are summarised according to quartile of dose length product

Quartile (DLP – mGy.cm)	I (<358.6)	II (358.6–431.6)	III (431.6–640.9)	IV (>640.9)	Correlation with DLP
BMI (kg/m^2^)	21.3 ± 3	24.2 ± 2	28 ± 3	32 ± 4.8	0.797**
Total adipose tissue (TAT)	5725 ± 3354	8834 ± 3136	11,554 ± 2883	17,813 ± 4864	0.832**
Muscle volume (MV)	7368 ± 1378	8033 ± 1585	10,329 ± 2428	11,818 ± 2219	0.660**
Bone volume (BV)	1197 ± 233	1375 ± 281	1602 ± 341	1605 ± 326	0.362**
Liver volume (LV)	1338 ± 309	1392 ± 357	1751 ± 423	1903 ± 495	0.507**
Right kidney volume (RKV)	127 ± 39	143 ± 36	169 ± 46	175 ± 47	0.393**
Left kidney volume (LKV)	130 ± 28	147 ± 41	169 ± 41	176 ± 46	0.399**
Spleen volume (SV)	191 ± 250	198 ± 144	259 ± 171	270 ± 205	0.142*

Data presented as mean ± standard deviation. Volumes represented as cm^3^

Spearman correlation co-efficient; **significant at the 0.001 level, *significant at the 0.05 level

A multiple linear regression was used to determine which body composition parameters were the strongest predictors of radiation dose. Patient dose length product was the dependent variable and total adipose, muscle, bone, liver, kidney and spleen volumes were included as independent variables in the regression model. An R^2^ value of 0.772 showed that the variables included in this model accounted for 77% of the variance of dose length product across our study sample. Total adipose volume was the strongest predictor of radiation dose in our group [B(95% CI) = 0.027 (0.024–0.030), *t* = 23.068, *p* < 0.001] and muscle volume also was a significant predictor of dose length product, albeit to a lesser degree [B(95% CI) = 0.022 (0.014–0.030), *t* = 13.516, p < 0.001]. Bone, liver, kidney and spleen volumes did not significantly predict patient dose length product in this regression model.

In subgroup analysis by sex and age, there was no significant difference between groups of different age (*p* = 0.101). We found that younger male patients had significantly less adipose tissue than younger females (*p* < 0.001) but no other significant differences were found upon comparison of adipose tissue volumes in other age and sex groups. Muscle volumes were significantly lower in female patients (*p* < 0.001). Bone volume was also significantly lower in female compared with male patients and younger female patients had significantly higher bone volumes than older female patients (p < 0.001). The differences in body composition between the groups are summarised in Table 2.

**Table 2:** Differences in DLP and body composition variables are summarised according to subgroup of patient age and sex

Subgroup	Younger female (<55.6 years)	Older female (>55.6 years)	Younger male (<55.6 years)	Older male (>55.6 years)
DLP (mGy.cm)	492 ± 250	452 ± 180	513 ± 235	620 ± 240
BMI (kg/m^2^)	25.1 ± 5.8	24.2 ± 4.8	27.4 ± 5	29.1 ± 4.5
Total adipose tissue (TAT)	10,603 ± 6719	10,861 ± 5200	9696 ± 5069	12,454 ± 5415
Muscle volume (MV)	8253 ± 2033	7996 ± 2028	10,346 ± 2793	11,092 ± 2313
Bone volume (BV)	1335 ± 214	1138 ± 226	1728 ± 306	1642 ± 273
Liver volume (LV)	1602 ± 453	1313 ± 360	1849 ± 471	1689 ± 438
Right kidney volume (RKV)	146 ± 43	128 ± 37	174 ± 38	169 ± 49
Left kidney volume (LKV)	148 ± 39	131 ± 37	176 ± 37	171 ± 45
Spleen volume (SV)	205 ± 229	151 ± 103	283 ± 227	287 ± 185

Data presented as mean ± standard deviation. Volumes represented as cm^3^

To determine which body composition parameter was the strongest predictor of radiation dose in each individual age-sex subgroup, the multiple linear regression model was repeated. Total adipose volume was again found to be the strongest predictor of dose length product (DLP) in each individual age-sex subgroup. Interestingly, muscle volume did not significantly predict dose length product values in younger female or younger male patients (*p* = 0.180 and *p* = 0.065 respectively) but was predictive of dose length product in older female and older male patients (Table 3).

**Table 3:** Multiple linear regression analysis with dose length product as dependent and body composition variables as the independent variables summarised according to patient group

**Group**	**All patients** (R^2^ = 0.772)		**Younger female** (R^2^ = 0.861)		**Older female** (R^2^ = 0.684)		**Younger male** (R^2^ = 0.758)		**Older male** (R^2^ = 0.778)	
Total adipose tissue (TAT)	**0.027**	**p < 0.001**	0.032	**p < 0.001**	**0.017**	**p < 0.001**	**−0.029**	**p < 0.001**	**9.094**	**p < 0.001**
Muscle volume (MV)	**0.022**	**p < 0.001**	0.014	p = 0.180	**0.030**	***p*** **= 0.011**	0.017	p = 0.065	**3.479**	***p*** **= 0.001**
Bone volume (BV)	0.043	*p* = 0.145	0.041	*p* = 0.557	0.091	*p* = 0.206	−0.031	*p* = 0.689	0.183	*p* = 0.855
Liver volume (LV)	0.032	*p* = 0.157	−0.038	*p* = 0.402	0.073	*p* = 0.258	0.050	*p* = 0.340	1.291	*p* = 0.201
Right kidney volume (RKV)	−0.141	*p* = 0.712	−0.617	*p* = 0.396	−0.119	*p* = 0.851	2.778	*p* = 0.477	−0.759	*p* = 0.451
Left kidney volume (LKV)	0.236	*p* = 0.562	1.197	p = 0.157	−0.097	*p* = 0.867	−1.614	*p* = 0.685	0.135	*p* = 0.893
Spleen volume (SV)	−0.017	*p* = 0.680	−0.011	*p* = 0.849	−0.399	*p* = 0.207	0.007	*p* = 0.951	−0.201	*p* = 0.842

Data presented are unstandardised co-efficient values (B) of the multiple linear regression model. Significance at the 0.05 level

BMI is a more readily obtainable parameter than total adipose tissue volume in clinical practice therefore we sought to determine the strength of association between patient BMI and the measured body composition variables in our cohort. We found that BMI correlated positively and significantly with all measured body composition parameters, in particular total adipose tissue (Spearman correlation = 0.798, *p* < 0.001) (Table 4). Stepwise linear regression using dose length product as the dependent and BMI and total adipose tissue as independent variables demonstrated however that total adipose tissue [B(95% CI) = 0.022 (0.019–0.025), *t* = 9.788, *p* < 0.001] is more predictive of dose length product than BMI [B(95% CI) = 16.045 (11.337–20.752), *t* = 6.681, p < 0.001].

**Table 4:** Radiation exposure and body composition variables are summarised according to quartile of patient body mass index

Quartile (BMI – kg/m^2^)	I (<22.7)	II (22.7–26.2)	III (26.2–29.3)	IV (>29.3)	Correlation with BMI
DLP (mGy.cm)	340 ± 68	420 ± 129	541 ± 158	804 ± 230	0.797**
Total adipose tissue (TAT)	6241 ± 3087	8863 ± 4083	12,068 ± 3374	17,316 ± 5185	0.798**
Muscle volume (MV)	7212 ± 1126	8828 ± 2277	10,044 ± 2664	11,644 ± 2006	0.624**
Bone volume (BV)	1196 ± 243	1417 ± 263	1585 ± 387	1600 ± 304	0.423**
Liver volume (LV)	1306 ± 374	1546 ± 382	1641 ± 422	1910 ± 480	0.524**
Right kidney volume (RKV)	131 ± 40	144 ± 30	160 ± 56	180 ± 43	0.394**
Left kidney volume (LKV)	136 ± 36	146 ± 27	162 ± 52	181 ± 44	0.398**
Spleen volume (SV)	187 ± 254	206 ± 133	266 ± 176	264 ± 201	0.132*

Data presented as mean ± standard deviation. Volumes represented as cm^3^

Spearman correlation co-efficient; **significant at the 0.001 level, *significant at the 0.05 level

Total adipose area measurements (VAA + SAA) obtained from a single slice (L4–L5 + 6 cm) correlated very closely with total adipose volume (Spearman’s correlation *r* = 0.978, p < 0.001). Stepwise linear regression using these two variables with dose length product as the dependent yielded almost identical results; total adipose volume [B(95% CI) = 0.018 (0.007–0.029), *t* = 2.746, *p* = 0.006]; VAA + SAA [B(95% CI) = 0.018 (0.005–0.031), *t* = 2.661, *p* = 0.008].

## Discussion

We conducted a descriptive, cross-sectional study in an attempt to identify the body composition predictors of radiation dose during abdominopelvic CT. Initial analysis confirmed that each individual variable measured including total abdominopelvic adipose, muscle, bone and solid organ volumes positively correlated, to varying degrees with dose length product. As each structure contributes to the overall x-ray attenuation of the abdomen/pelvis, a positive correlation for each individual parameter was also not surprising given that patients with larger adipose, organ, bone and muscle volumes had a larger body habitus in our group. Linear regression analysis was then used to determine which body composition parameter was the strongest predictor of radiation dose. We found that total adipose volume was the strongest predictor and that muscle volume was also a significant but weaker predictor of dose length product. Adipose tissue is less dense and therefore less attenuating than all other body composition parameters measured in our study but its variability across our cohort of patients was large. Patients in the fourth quartile of dose length product (DLP > 620 mGy.cm) had a mean total adipose volume greater than three times more than patients who were in the first quartile of dose length product (DLP < 358 mGy.cm). In comparison, patients in the fourth quartile of dose length product had a mean muscle volume that was approximately 1.5 times more than patients who were in the first quartile of dose length product. We divided our study sample into four age- and sex-stratified groups and found that total adipose volume remained the strongest predictor of dose length product in each subgroup and interestingly that muscle volumes were predictive of dose length product in older patients only.

Our findings therefore advance previous knowledge that has established that patients with an elevated body weight [6], body mass index [4] or cross-sectional area [5, 7] receive larger doses of ionising radiation during abdominopelvic CT with ATCM. Our results indicate that, of the many body tissues that constitute a patient’s BMI or cross-sectional area, it is a patient’s total abdominal/pelvic adipose tissue that is the strongest predictor of radiation dose during abdominopelvic CT.

The challenges that obese patients present to radiology departments, particularly in relation to dose reduction, are now increasingly recognised [15] because more than 30% of US adults and almost 20% of European adults have a body mass index of greater than 30 kg/m^2^ [16, 17]. Although the shielding effects of visceral and subcutaneous adipose tissue result in over 50% less radiation dose to organs deep within the abdomen in morbidly obese individuals [18], ATCM results in logarithmic increases in tube current with increasing patient size [7] and abdominopelvic organ doses in obese patients are consequentially higher [6]. Furthermore, an effective dose to the skin and more superficial organs is proportionally increased with increasing patient size because of the increased tube current and reduced distance between the patient and the x-ray source [18].

The necessity to optimise and tailor the ATCM technique has long been recognised in patients with a large body habitus [5]. Such efforts to refine dose and noise index parameters may be of increased importance as new dose reduction technologies such as iterative reconstruction are being used with success in patients with a large body habitus [19, 20]. Weight-based optimisation of ATCM protocols has the advantage of being easily executed but may not provide an ideal basis to reduce dose as short obese patients may have the same weight as tall thin patients [6]. Our results suggest that quantification of total abdominal adipose tissue could potentially guide radiation dose reduction pathways and facilitate individualised protocols for optimisation of the ATCM protocol, as it appears to be the strongest body composition predictor of dose length product at abdominopelvic CT. This process would be greatly facilitated if robust automated quantification of total abdominal adipose tissue could be performed. A possible approach would be to acquire a single representative abdominal CT section from which an automated quantification of total abdominal adipose tissue could be performed. Another possibility in the case of follow-up or repeat CT studies would be to analyse previous CT scans, to allow quantification of total abdominal adipose tissue and allow protocols to be modelled based on abdominal fat distribution, thereby optimising the dose while preserving image quality. It should be noted that measurement of visceral adipose tissue appears reproducible between various dose reduction and image reconstruction strategies [21].

We concede that our study is entirely descriptive with studies from a single CT scanner and that the practical relevance of our results will require further assessment and research. To limit possible confounding factors we used a single scanner with a standard imaging technique and we acknowledge that newer dose reduction techniques and scanner technologies (such as automated tube voltage selection) may impart differing doses to the various tissue types while reducing the overall dose [22]. We acknowledge that the volume of intravenous and oral contrast administered affects automated tube current modulation [6, 23] and our cohort received standard IV and oral contrast, not weight-adapted doses of contrast. Oral contrast distribution was heterogeneous throughout the gastrointestinal tract, as completely homogenous bowel opacification is difficult to attain and may impart a dose length product increase that would be greater than a single small organ such as a single kidney or the spleen.

A practical limitation of our study is that quantitatively obtaining a patient’s total adipose volume represents an additional workflow challenge in the day-to-day practice of CT. CT scanners could also benefit practically from including the patient’s height in addition to their weight to optimise the radiation dose, as short obese patients may have the same weight as tall thin patients [6].

We did not formally record the time taken to perform the quantitative measurements in this study but manual delineation of organs and the process of bone and muscle segmentation were subjectively laborious and time consuming, taking at least 20 min per patient. In contrast the process of Hounsfield unit thresholding to generate total adipose measurements across each series was subjectively quick (~30 s) and interobserver agreement was highest for adipose assessment in our study (intraclass correlation of TAT = 0.999, *p* < 0.001, *n* = 71), suggesting that total adipose estimation from a patient’s previous CT scan would be more feasible than other estimates in clinical practice. However, obtaining a total adipose volume would also be impossible in patients who are undergoing their first CT scan. A consideration in this situation would be to analyse adipose area on a single CT image taken at the time of the topogram. Many studies investigating body composition using cross-sectional images have used this approach particularly when estimating visceral and subcutaneous adipose quantities with good success [13, 14]. The excess radiation dose imparted during the acquisition of this single slice has been estimated to be in the range of 0.015–0.019 mSv/37–54 kg of patient weight, which compares favourably with the typical dose imparted during the acquisition of a CT topogram. In our patient group total adipose area measurements (VAA + SAA) obtained from a single slice (L4-L5 + 6 cm) correlated very well with total adipose volume (Spearman’s correlation *r* = 0.978, *p* < 0.001), which may be of value in the practical application of our findings.

In conclusion, the abdomen and pelvis house many organs and tissues of varying volume and density but the volume of adipose tissue was the strongest predictor of radiation dose. This finding is fortunate however as during the interpretation of CT images, intra-abdominal fat, when present in sufficient volume, can separate bowel loops from adjacent intra- and retroperitoneal structures effectively providing “internal” contrast that can help the interpreting radiologist to more accurately localise and identify pathology. As a consequence, numerous studies suggest that the decision to administer or withhold oral contrast agents should be based on the patient’s adiposity [24–26]. The authors raise the possibility that more aggressive dose reduction could also be employed on the basis of adipose tissue volumes that could be derived from previous CT scans or from single-slice analysis in those without previous CTs. Further research is required to evaluate the practical relevance of these parameters and to better define how it may help to optimise the CT technique and reduce the radiation dose.
